# Phylogenomics and Coalescent Analyses Resolve Extant Seed Plant Relationships

**DOI:** 10.1371/journal.pone.0080870

**Published:** 2013-11-21

**Authors:** Zhenxiang Xi, Joshua S. Rest, Charles C. Davis

**Affiliations:** 1 Department of Organismic and Evolutionary Biology, Harvard University, Cambridge, Massachusetts, United States of America; 2 Department of Ecology and Evolution, Stony Brook University, Stony Brook, New York, United States of America; University of California, Berkeley, United States of America

## Abstract

The extant seed plants include more than 260,000 species that belong to five main lineages: angiosperms, conifers, cycads, *Ginkgo*, and gnetophytes. Despite tremendous effort using molecular data, phylogenetic relationships among these five lineages remain uncertain. Here, we provide the first broad coalescent-based species tree estimation of seed plants using genome-scale nuclear and plastid data By incorporating 305 nuclear genes and 47 plastid genes from 14 species, we identify that i) extant gymnosperms (i.e., conifers, cycads, *Ginkgo*, and gnetophytes) are monophyletic, ii) gnetophytes exhibit discordant placements within conifers between their nuclear and plastid genomes, and iii) cycads plus *Ginkgo* form a clade that is sister to all remaining extant gymnosperms. We additionally observe that the placement of *Ginkgo* inferred from coalescent analyses is congruent across different nucleotide rate partitions. In contrast, the standard concatenation method produces strongly supported, but incongruent placements of *Ginkgo* between slow- and fast-evolving sites. Specifically, fast-evolving sites yield relationships in conflict with coalescent analyses. We hypothesize that this incongruence may be related to the way in which concatenation methods treat sites with elevated nucleotide substitution rates. More empirical and simulation investigations are needed to understand this potential weakness of concatenation methods.

## Introduction

**Figure 1:**
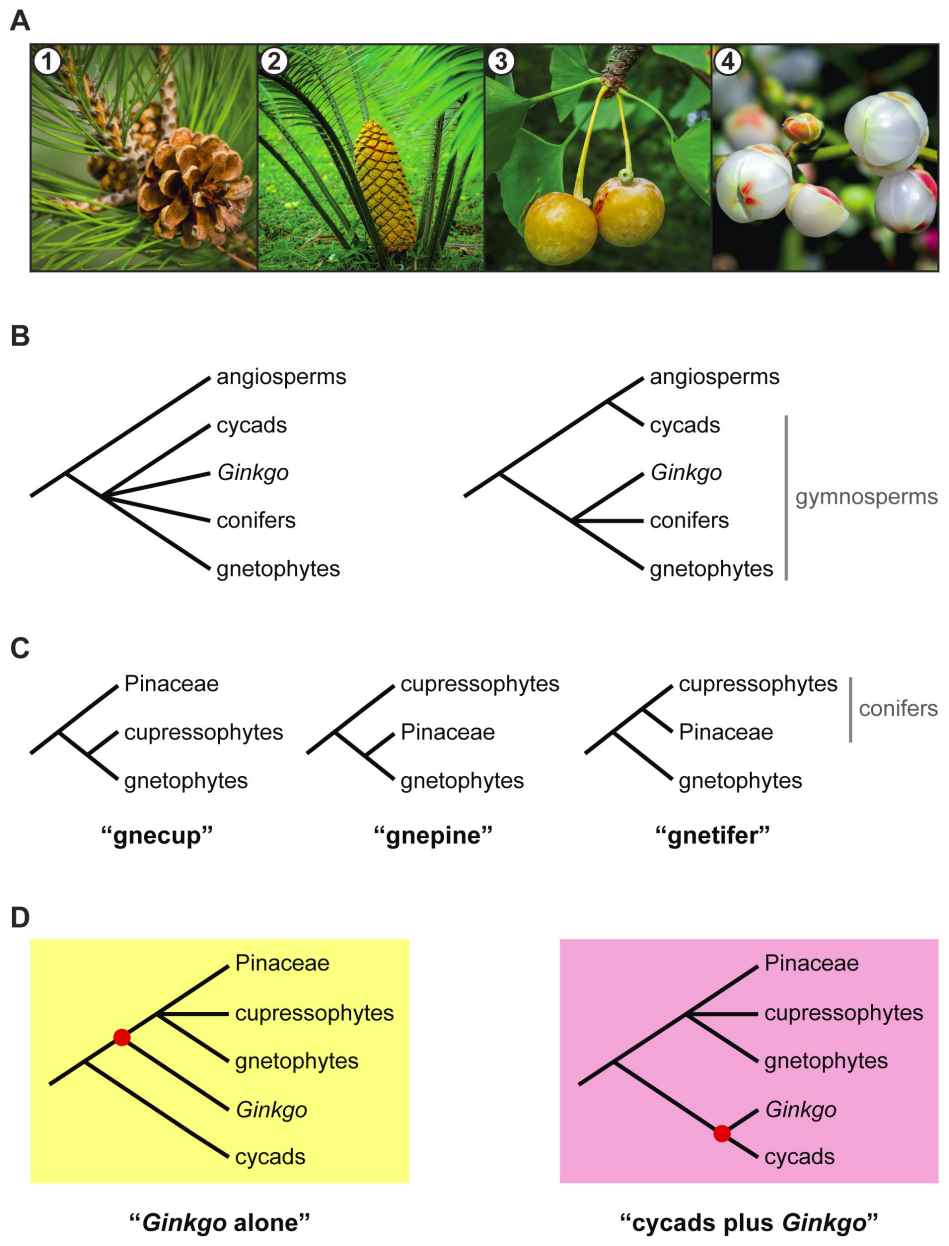
Conflicting phylogenetic relationships among extant gymnosperms. (A) The four main lineages of extant gymnosperms: (1) conifers (*Pinus resinosa*), (2) cycads (*Cycas* *sp.*), (3) *Ginkgo biloba*, and (4) gnetophytes (*Ephedra chilensis*). (B) Two main hypotheses for phylogenetic relationships of gymnosperms. (C) Three main hypotheses for the phylogenetic placement of gnetophytes. (D) Two main hypotheses for the phylogenetic placement of *Ginkgo*.

Seed plants originated at least 370 million years ago [1] and include more than 260,000 extant species [2], making them the most species rich land plant clade. These species are placed in five main lineages: angiosperms, conifers, cycads, *Ginkgo*, and gnetophytes [3]. By far the greatest species diversity is found in the angiosperms; the remaining four lineages constitute the extant gymnosperms (Figure 1A), meaning “naked seeds”. Today’s gymnosperms are a shadow of their former glory–only ~1,000 species currently exist [2]. Nevertheless, they are of huge ecological and economic importance, especially for their timber and horticultural value.

Despite tremendous efforts to resolve phylogenetic relationships among the five extant seed plant lineages using molecular data, these relationships remain uncertain. For example, early studies identified the monophyly of extant gymnosperms [4-11], but more recent studies using duplicate gene rooting have suggested that cycads are instead more closely related to angiosperms than they are to other extant gymnosperms (Figure 1B) [3,12]. Similarly, the gnetophytes, which were previously thought to be sister to angiosperms based on morphological characters (i.e., the anthophyte hypothesis; [13,14]), are now grouped with other extant gymnosperms using molecular data. Establishing the phylogenetic placement of gnetophytes among extant gymnosperms, however, remains problematic. Recent molecular studies have suggested three conflicting hypotheses of gnetophyte relationships: the gnecup (i.e., gnetophytes sister to cupressophytes; [9,15]), gnepine (i.e., gnetophytes sister to Pinaceae; [7,8,10,16-24]), and gnetifer (i.e., gnetophytes sister to conifers; [5,25]) hypotheses (Figure 1C). In addition, early studies concatenating multiple genes placed *Ginkgo* alone as sister to conifers and gnetophytes within the extant gymnosperm clade [7-11,16-18,26-28]. However, more recent studies using additional genes have suggested that a clade containing cycads plus *Ginkgo* cannot be excluded as sister to all remaining extant gymnosperms (Figure 1D) [15,19,21-24,29,30]. In particular, attempts to include data that are less prone to saturation due to high rates of substitution (e.g., amino acid sequences and slow-evolving nucleotide sequences) have lead to increasing support for the placement of cycads plus *Ginkgo* as sister to all remaining extant gymnosperms [15,21,23,24]. For all of these reasons, a broader comparative phylogenomic assessment of these questions is warranted to better understand the evolution of extant seed plants.

Advances in next-generation sequencing and computational phylogenomics represent tremendous opportunities for inferring species relationships using hundreds, or even thousands, of genes. Until now the reconstruction of broad seed plant phylogenies from multiple genes has relied almost entirely on concatenation methods [7-11,15-19,21,23,24,29,31-37], in which phylogenies are inferred from a single combined gene matrix [38]. These analyses assume that all genes have the same, or very similar, evolutionary histories. Theoretical and simulation studies, however, have shown that concatenation methods can yield misleading results, especially if gene trees are highly heterogeneous [39-43]. In contrast, recently developed coalescent-based methods estimate the species phylogeny from a collective set of gene trees, which permit different genes to have different evolutionary histories [44-46]. Both theoretical and empirical studies have shown that coalescent methods can better accommodate gene heterogeneity [44-48].

Here, our phylogenomic analyses of 14 species represent the first coalescent-based species tree estimation of seed plants. By incorporating hundreds of nuclear genes as well as a full complement of plastid genes, we also provide a direct comparison of phylogenetic relationships inferred from nuclear and plastid genomes.

## Results and Discussion

### Taxon and gene sampling of nuclear and plastid genes

Our nuclear gene taxon sampling included 12 species representing all major lineages of extant seed plants (i.e., angiosperms [*Amborella trichopoda* and *Nuphar advena*], conifers [*Cryptomeria japonica*, *Picea glauca*, *Picea sitchensis*, *Pinus contorta*, and *Pinus taeda*], cycads [*Cycas rumphii* and *Zamia furfuracea*], *Ginkgo biloba*, and gnetophytes [*Gnetum gnemon* and *Welwitschia mirabilis*]) [3]. One fern (*Adiantum capillus-veneris*) and one lycophyte (*Selaginella moellendorffii*) were included as outgroups (Table 1). Of these 14 species, the coding sequences of *Selaginella* were obtained from a whole-genome sequencing project, and the rest were from deeply sequenced transcriptomes that each included at least 6,000 assembled unigenes. Using a Markov clustering algorithm [49], the 234,040 protein-coding sequences (sequences with in-frame stop codons or shifted reading frames were excluded prior to clustering) from these 14 species were grouped into 14,215 gene clusters, of which 496 passed our initial criteria for establishing low-copy nuclear genes as described in the Materials and Methods section. Following this initial filter, the average numbers of sequences and species for each gene cluster were ten and eight, respectively. Additionally, of these 496 gene clusters, 305 remained following our paralogue pruning filter (see Materials and Methods), and the average number of species and sites for each gene cluster were nine and 509, respectively (Table S1). The final concatenated nuclear gene matrix included 155,295 nucleotide sites and 37.1% missing data (including gaps and undetermined characters).

**Table 1:** Data sources of nuclear gene sequences included in our phylogenetic analyses.

**Species**	**Sources**	**No. of coding sequences used in clustering**	**No. of sequences used in phylogenetic analyses**	**Average GC-content**
*Adiantum capillus-veneris*	[50]	5,724	107	47.1%
*Amborella trichopoda*	[51]	32,987	251	45.1%
*Cryptomeria japonica*	[50]	8,224	184	44.0%
*Cycas rumphii*	[50]	4,211	118	45.1%
*Ginkgo biloba*	[50]	3,739	88	44.7%
*Gnetum gnemon*	[50]	2,016	44	44.8%
*Nuphar advena*	[51]	68,266	266	48.1%
*Picea glauca*	[50]	23,693	288	44.7%
*Picea sitchensis*	[50]	13,298	283	44.9%
*Pinus contorta*	[50]	7,844	260	44.5%
*Pinus taeda*	[50]	28,670	271	44.8%
***Selaginella moellendorffii***	[52]	21,094	305	54.3%
*Welwitschia mirabilis*	[50]	3,170	80	43.9%
*Zamia vazquezii*	[51]	11,104	214	45.0%

Species with sequenced genome is highlighted in bold.

To compare the evolutionary history between nuclear and plastid genomes, we obtained the annotated plastid genomes from 12 seed plants (i.e., angiosperms [*Amborella trichopoda* and *Nuphar advena*], conifers [*Cryptomeria japonica*, *Picea abies*, *Picea morrisonicola*, *Pinus koraiensis*, and *Pinus taeda*], cycads [*Cycas revoluta* and *Zamia furfuracea*], *Ginkgo biloba*, and gnetophytes [*Gnetum parvifolium* and *Welwitschia mirabilis*]), plus one fern (*Adiantum capillus-veneris*) and one lycophyte (*Selaginella moellendorffii*) as outgroups (Table 2). These 14 species represent the same taxonomic placeholders as those in our nuclear gene analyses. The 685 protein-coding sequences from the 14 plastid genomes were grouped into 59 gene clusters, of which 47 remained following the filtering criteria described above. The average number of species and sites for these 47 gene clusters were 12 and 1,063, respectively (Table S2). The final concatenated plastid gene matrix included 49,968 nucleotide sites and 14.1% missing data.

**Table 2:** Data sources of plastid gene sequences included in our phylogenetic analyses.

**Species**	**GenBank accession number**	**No. of sequences used in phylogenetic analyses**	**Average GC-content**
*Adiantum capillus-veneris*	NC_004766	46	42.8%
*Amborella trichopoda*	NC_005086	44	40.1%
*Cryptomeria japonica*	NC_010548	46	38.0%
*Cycas revoluta*	NC_020319	47	40.3%
*Ginkgo biloba*	NC_016986	47	40.4%
*Gnetum parvifolium*	NC_011942	33	38.6%	
*Nuphar advena*	NC_008788	44	40.6%
*Picea abies*	NC_021456	36	40.7%
*Picea morrisonicola*	NC_016069	35	40.7%
*Pinus koraiensis*	NC_004677	36	40.5%
*Pinus taeda*	NC_021440	36	40.4%
*Selaginella moellendorffii*	NC_013086	47	50.8%
*Welwitschia mirabilis*	NC_010654	32	37.2%
*Zamia furfuracea*	JQ770198-JQ770303	32	41.4%

### Inferring Species Relationships Using Coalescent and Concatenation Methods

Species relationships were first estimated from nucleotide sequences using the recently developed coalescent method: Species Tree Estimation using Average Ranks of Coalescence (STAR) [46]. Since this method is based on summary statistics calculated across all gene trees, a small number of outlier genes that significantly deviate from the coalescent model have relatively little effect on the accurate inference of the species tree [48]. We note that while all plastid genes are generally expected to share the same history, evidence of recombination, heteroplasmy, and incomplete lineage sorting in plastid genomes suggests that this may not always apply (e.g., 53-57). Thus, we additionally analyzed plastid genes using the coalescent method. We compared the results from coalescent analyses of both nuclear and plastid genes with those from concatenation analyses using maximum likelihood (ML) as implemented in RAxML [58]. Statistical confidence was established for both methods using a multilocus bootstrapping approach [59], in which genes were resampled with replacement followed by resampling sites with replacement within each gene.

**Figure 2:**
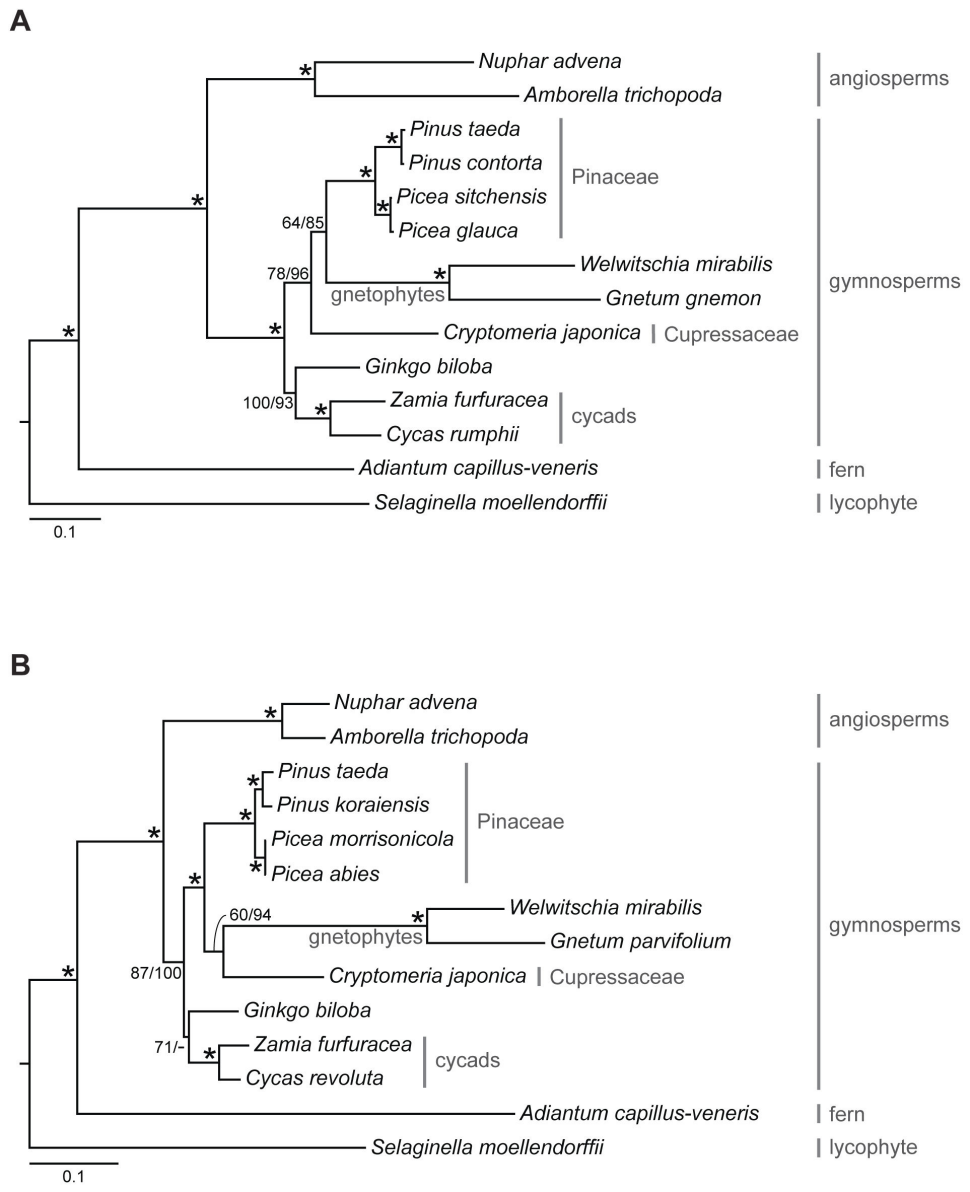
Species trees inferred from (A) 305 nuclear genes and (B) 47 plastid genes using the coalescent method (STAR). Bootstrap percentages (BPs) from STAR/RAxML are indicated above each branch; an asterisk indicates that the clade is supported by 100 BPs from both STAR and RAxML. Branch lengths were estimated by fitting the concatenated matrices to the inferred topology from STAR.

 Our species trees inferred from coalescent and concatenation methods largely agree with each other (Figure 2). Similarly, analyses of nuclear and plastid genes are largely in agreement. All analyses strongly support (≥87 bootstrap percentage [BP]) the monophyly of extant gymnosperms. The lone placement that shows conflict between the nuclear and plastid gene trees is for the gnetophytes (i.e., *Gnetum* and *Welwitschia*). Our coalescent and concatenation analyses of nuclear genes support the gnepine hypothesis (i.e., gnetophytes sister to Pinaceae [*Picea* and *Pinus*]) with 64 BP and 85 BP, respectively (Figure 2A). In contrast, our coalescent and concatenation analyses of plastid genes support the gnecup hypothesis (i.e., gnetophytes sister to cupressophytes [Cryptomeria]) with 60 BP and 94 BP, respectively (Figure 2B). Moreover, in each of these cases the rival topology is rejected using the approximately unbiased (AU) test [60]: the gnecup placement is rejected for concatenated nuclear gene matrix (*p*-value = 0.001) and the gnepine placement is rejected for concatenated plastid gene matrix (*p*-value = 0.001). This conflicting placement between the nuclear and plastid genomes is consistent with previous studies (e.g., 15,19,22), although our study is a direct comparison using a similar set of species for both genomes. These results suggest that the nuclear and plastid genomes of gnetophytes may have distinctly different evolutionary histories.

An additional well-supported placement we uncovered here relates to cycads and *Ginkgo*. Our coalescent and concatenation analyses of nuclear genes strongly support (100 BP and 93 BP, respectively) cycads (i.e., *Cycas* and *Zamia*) plus *Ginkgo* as sister to all remaining extant gymnosperms (Figure 2A and see red dots in Figure 1D for clades under consideration). The rival placement of *Ginkgo* alone as sister to conifers and gnetophytes (i.e., the “*Gingko* alone” hypothesis) is rejected for the concatenated nuclear gene matrix (*p*-value = 0.004, AU test). In addition, our coalescent analyses of plastid genes similarly support (71 BP) the monophyly of cycads plus *Ginkgo* (Figure 2B). The concatenation analyses of plastid genes, in contrast, weakly support (56 BP) the “*Gingko* alone” hypothesis.

Because sequences from both cycads and *Ginkgo* were not present in all 305 nuclear genes, we conducted an additional analysis using only those genes that included both cycads and *Ginkgo* (sequences from both cycads and *Ginkgo* were present in all 47 plastid genes; see Table 2). This allows us to test if the phylogenetic placement of *Ginkgo* inferred from nuclear genes is sensitive to missing data. Although the number of nuclear gene clusters declines to 69 when applying this taxon filter, the results are identical to those above: the coalescent and concatenation analyses strongly support (95 BP and 97 BP, respectively) cycads plus *Ginkgo* as sister to all remaining extant gymnosperms.

**Figure 3:**
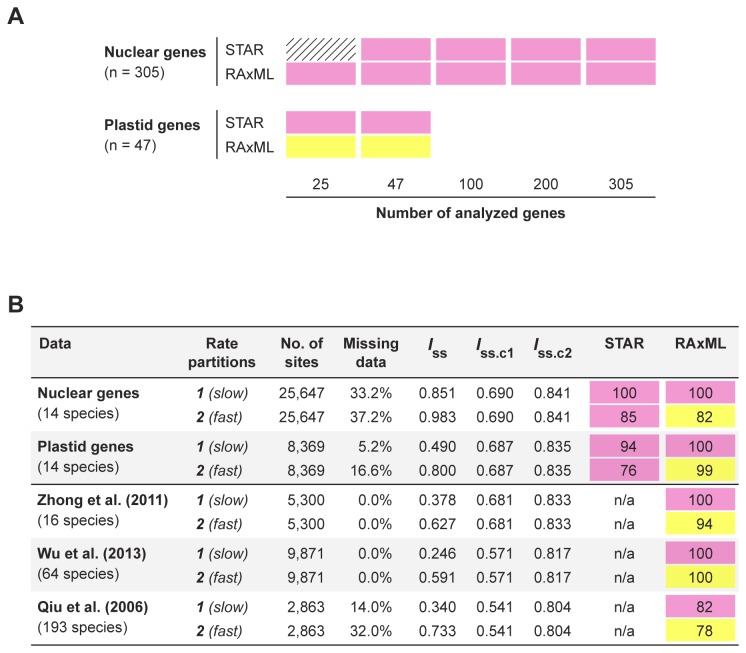
Summary of bootstrap percentages (BPs) from coalescent and concatenation analyses using different gene subsampling and rate partitions. (A) BPs from coalescent and concatenation analyses using different gene subsampling. The 305 nuclear genes were subsampled for four different gene size categories (i.e., 25, 47, 100, or 200 genes; 10 replicates each), and the 47 plastid genes were subsampled for 25 genes (10 replicates). Cells with hatching indicate that support for the placement of *Ginkgo biloba* from all replicates is below 80 BP; colored cells indicate relationships that received bootstrap support ≥80 BP from at least one replicate (pink = cycads plus *Ginkgo* as sister to all remaining extant gymnosperms, yellow = *Ginkgo* alone as sister to conifers and gnetophytes within extant gymnosperms; see also Figure 1D). (B) BPs from coalescent and concatenation analyses across different nucleotide rate partitions. Parsimony informative sites in concatenated matrices were sorted based on estimated evolutionary rates, and subsequently divided into two equal partitions. The index of substitution saturation (*I*_SS_) was used to measure nucleotide substitution saturation for sites within each rate partition. The two critical *I*_SS_ values, i.e., *I*_SS.C1_ and *I*_SS.C2_, were estimated using an asymmetrical and symmetrical topology, respectively (for data including more than 32 species, only values estimated from 32 terminals are shown here).

To further investigate if the placement of *Ginkgo* is sensitive to the number of sampled genes, we randomly subsampled the 305 nuclear genes in four different gene size categories (i.e., 25, 47, 100, or 200 genes; 10 replicates each). We similarly subsampled the 47 plastid genes (i.e., 25 genes with 10 replicates). Even as the sample size declines, the coalescent and concatenation analyses of nuclear genes strongly support (≥80 BP) cycads plus *Ginkgo* as sister to all remaining extant gymnosperms. Support for this relationship only dropped below 80 BP when the number of subsampled nuclear genes was 25 for the coalescent analyses (Figure 3A). For the 25 subsampled plastid genes, the coalescent analyses also support cycads plus *Ginkgo* with ≥80 BP. In contrast, concatenation analyses of 25 subsampled plastid genes support the “*Gingko* alone” hypothesis with ≥80 BP (Figure 3A). Thus, our results are robust to the number of genes sampled, including the discordant placements of *Ginkgo* between coalescent and concatenation analyses of plastid genes.

### Accommodating rate heterogeneity in coalescent and concatenation analyses

Despite the fact that our coalescent and concatenation analyses largely agree with each other, we are interested in exploring the influence of nucleotide substitution rates on phylogenetic inference of seed plant relationships. It has long been appreciated that elevated rates of molecular evolution can lead to multiple substitutions at the same site [61,62], which can be especially misleading for resolving deeper relationships if the substitution model fails to correct for high levels of saturation in fast-evolving sites [24,62-68]. This is especially relevant for inferring the phylogeny of early diverging gymnosperms given their ancient origin [69-72]. Here, to assess the effect of rate heterogeneity, we partitioned nucleotide sites in our concatenated matrices according to estimated evolutionary rates.

The relative evolutionary rate of each site in our concatenated matrices was estimated using the Observed Variability (OV) method [62], which compares all sequences at a given site in a pair-wise manner, and uses the total number of mismatches between species as the measure of site variability. Importantly, since the OV is a tree-independent approach, it is free from systematic bias of estimating evolutionary rates using an inaccurate phylogeny [62]. We sorted all parsimony informative sites in our concatenated nucleotide matrices based on their relative evolutionary rates and then divided them into two equal partitions (Figures S1A and S1B). For nuclear genes each rate partition contains 25,647 sites, and for plastid genes each partition contains 8,369 sites.

When analyzing data from each rate partition separately, the coalescent method supports (≥76 BP) cycads plus *Ginkgo* as sister to all remaining extant gymnosperms across all rate partitions for both nuclear and plastid genes (Figure 3B). In contrast, the concatenation method produces well supported, but incongruent results, across different rate partitions (Figure 3B). Here, the slow-evolving sites corroborate results from our coalescent analyses and place cycads sister to *Ginkgo* with 100 BP for both nuclear and plastid genes. However, fast-evolving sites support the “*Gingko* alone” hypothesis with 82 BP and 99 BP for nuclear and plastid genes, respectively. Additionally, when the placement of cycads plus *Ginkgo* is inferred using the concatenation method, the rival placement of “*Ginkgo* alone” is rejected (*p*-value < 0.001, AU test). Similarly, in all cases when “*Ginkgo* alone” is supported, the rival placement of cycads plus *Ginkgo* is rejected (*p*-value < 0.001, AU test).

To determine if nucleotide substitution saturation might influence the incongruent placements of *Ginkgo* in our concatenation analyses, we characterized sites within each of our rate partitions using an entropy-based index of substitution saturation (*I*_SS_) [73]. As *I*_SS_ approaches 1, or if *I*_SS_ is not smaller than the critical *I*_SS_ value (*I*_SS.C_), then sequences are determined to exhibit substantial saturation [73]. Our analyses demonstrate that for plastid genes (Figure 3B), the slow-evolving sites exhibit no evidence of saturation (i.e., *I*_SS_ is significantly smaller than *I*_SS.C_; *p*-value < 0.001, two-tailed *t*-test), while the fast-evolving sites show evidence of substantial saturation (i.e., *I*_SS_ is greater than *I*_SS.C_ when the true topology is asymmetrical). In contrast, our analyses indicate that all rate partitions for nuclear genes show evidence of substantial saturation, but the slow-evolving sites exhibit lower overall levels of saturation (Figure 3B). Thus, the nuclear and plastid genes together suggest that the incongruence we observe in the placement of *Ginkgo* across rate partitions using the concatenation method may be related to higher overall levels of substitution saturation in fast-evolving nucleotide sites. Further exploration of this question is warranted.

Finally, since previous studies have established the importance of taxon sampling in determining the placement of *Ginkgo* [15], we re-analyzed three concatenated nucleotide matrices from previous studies to confirm that our results are not biased by insufficient taxon sampling. These three matrices include a wide breadth of taxon and gene sampling: i) 16 seed plants using 52 plastid genes from Zhong et al. [24], ii) 64 vascular plants using 53 plastid genes from Wu et al. [15], and iii) 193 green plants using six genes representing all three plant genomic compartments (i.e., nucleus, plastid, and mitochondrion) from Qiu et al. [29]. Our phylogenetic analyses of these three matrices mirror the results using the concatenation method summarized above. When including only those slow-evolving sites identified by the OV method (Figures S1C–S1E), the clade containing cycads plus *Ginkgo* is well supported (≥82 BP; Figure 3B). In contrast, analyzing only the fast-evolving sites supports (≥78 BP) the “*Gingko* alone” hypothesis (Figure 3B). Importantly, the slow-evolving sites in all three matrices exhibit no evidence of saturation (*p*-value < 0.001, two-tailed *t*-test); while the fast-evolving sites in two of three matrices show evidence of substantial saturation (Figure 3B).

## Conclusions

 Our phylogenomic analyses of seed plants identify three main results: i) extant gymnosperms are monophyletic, ii) gnetophytes exhibit discordant placements within conifers between their nuclear and plastid genomes, and iii) cycads plus *Ginkgo* form a clade that is sister to all remaining extant gymnosperms. Our results also show that standard concatenation analyses of both nuclear and plastid genes produce well supported, but conflicting placements of key taxa across sites with different substitution rates. Determining the causes of this incongruence, however, requires more empirical and simulation studies. Here, we hypothesize that this incongruence may be related to the way in which concatenation methods treat sites with elevated nucleotide substitution rates. Although our concatenation analyses of fast-evolving nucleotide sites produced the “*Ginkgo* alone” topology, the signal from slow-evolving sites appears to have prevailed. Thus, we did not observe strongly conflicting placements of *Ginkgo* between coalescent and concatenation methods when analyzing all sites together. One interpretation of these results is that concatenation analyses of full data sets may not be heavily misled by a subset of sites with elevated substitution rates. However, an extrapolation of our specific results suggests that as saturated sites increase in phylogenomic data sets, standard concatenation methods may produce strongly supported but incorrect results. In contrast, coalescent analyses of the same data sets demonstrated consistent placement of cycads plus *Ginkgo*, suggesting that coalescent-based methods better deal with rate heterogeneity [44-48].

How does this increased phylogenetic resolution enhance our understanding of seed plant evolution? Cycads and *Ginkgo* share a number of morphological characters, such as their unusual pattern of pollen tube development [74], flagellated male gametes [75,76], simple female strobili [77], and embryo development [78]. In light of the increasing support of cycads plus *Ginkgo* we identify here, some of these traits, which have been commonly thought to be symplesiomorphies of gymnosperms [13,78], may actually represent synapomorphies of the cycads plus *Ginkgo* clade [15]. Assessing these questions going forward will be challenging, however, given the phenomenally high rate of extinction suffered by gymnosperms [79]. A thoughtful assessment of this question is only likely to be answered with more exhaustive sampling of fossil lineages.

## Materials and Methods

### Data acquisition and sequence translation

Gene sequences from both nuclear and plastid genomes were gathered for this study. For nuclear genes, assembled unique transcripts were obtained (Table 1) and then translated to amino acid sequences using prot4EST v2.2 [80]. For plastid genes, the fully annotated plastid genomes were obtained from NCBI GenBank (Table 2).

### Homology Assignment and Sequence Alignment

The establishment of sequence homology for phylogenetic analyses followed Dunn et al. [81] and Hejnol et al. [82]. Briefly, sequence similarity was first assessed for all amino acid sequences using BLASTP v2.2.25 [83] with 10^-20^ *e*-value threshold, and then grouped using a Markov cluster algorithm as implemented in MCL v09-308 [49] with the inflation value equals 5.0. Clusters were required to i) include at least one sequence from *Selaginella* (for outgroup rooting), ii) include sequences from at least four species, iii) include at least 100 amino acids for each sequence [84], iv) have a mean of less than five sequences per species, and v) have a median of less than two sequences per species. Amino acid sequences from each cluster were aligned using MUSCLE v3.8.31 [85], and ambiguous sites were trimmed using trimAl v1.2rev59 [86] with the heuristic automated method. Sequences were removed from the alignment if they contained less than 70% of the total alignment length [87]. Nucleotide sequences were then aligned according to the corresponding amino acid alignments using PAL2NAL v14 [88]. For each cluster, the gene tree was inferred from nucleotide alignments using RAxML v7.2.8 with the GTRGAMMA substitution model. All but one sequence were deleted in clades of sequences derived from the same species, i.e., monophyly masking, using Phyutility v2.2.6 [89].

### Paralogue pruning and species tree assessment

Paralogue pruning of each gene tree used for species tree assessment followed Hejnol et al. [82]. Briefly, we first identified the maximally inclusive subtree that contains no more than one sequence per species. This subtree is then pruned away and the remaining tree is used as a substrate for another round of pruning. The process is repeated until the remaining tree has no more than one sequence per species. Subtrees produced by paralogue pruning were then filtered to include only those with i) seven or more species and ii) 60% of the species present in the original cluster from which they were derived.

For the coalescent approach, individual gene trees were first inferred using RAxML with the GTRGAMMA substitution model from nucleotide sequences, species relationships were then estimated from gene trees using STAR as implemented in Phybase v1.3 [90]. For concatenation analyses, the concatenated nucleotide matrix was generated from individual genes using Phyutility, and the best-scoring ML tree was obtained using RAxML with the GTRGAMMA substitution model. Bootstrap support was estimated for both coalescent and concatenation methods using a multilocus bootstrap approach as described in the Results and Discussion section with 200 replicates.

Alternative topology tests were performed in the ML framework using the AU test as implemented in scaleboot v0.3-3 [91]. All constrained searches were conducted in RAxML using the GTRGAMMA substitution model.

### Gene subsampling

To subsample gene clusters, the 305 nuclear gene clusters were randomly selected for the sizes of 25, 47, 100, and 200 genes, and the 47 plastid gene clusters were randomly selected for the size of 25 genes. Ten sets of gene clusters were selected as replicates for each size. Species trees and bootstrap support were estimated using STAR and RAxML for each replicate as described above.

### Estimation of evolutionary rate and substitution saturation assessment

The OV method was used to measure the relative evolutionary rate of each site in all five concatenated matrices (Figure 3B) as described in the Results and Discussion section. Species trees and bootstrap supports were estimated using STAR and RAxML for each rate partition as described above.

Nucleotide substitution saturation was measured using *I*_SS_ as implemented in DAMBE [92]. *I*_SS_ was estimated for each rate partition from 200 replicates with gaps treated as unknown states.

## Supporting Information

Figure S1 **The estimated evolutionary rates for nucleotide sites in all five concatenated matrices analyzed in this study.** Parsimony informative sites in each concatenated matrix were sorted based on the Observed Variability (OV) method, and subsequently divided into two equal partitions.
(PDF)

Table S1 **Data characteristics for all 305 nuclear genes, including the locus ID of sequence from *Selaginella moellendorffii* in each gene, number of species per gene, number of nucleotide sites per gene, and percentage of gaps per gene.**
(PDF)

Table S2 **Data characteristics for all 47 plastid genes, including number of species per gene, number of nucleotide sites per gene, and percentage of gaps per gene.**
(PDF)
