# Supplementary material for: Phylogenomics and Coalescent Analyses Resolve Extant Seed Plant Relationships
Source: PLoS One. 2013 Nov 21;8(11):e80870. doi: 10.1371/journal.pone.0080870 (PMC3836751; doi:10.1371/journal.pone.0080870)

Figure S1. The estimated evolutionary rates for nucleotide sites in all five concatenated matrices analyzed in this study. Parsimony informative sites in each concatenated matrix were sorted based on the Observed Variability (OV) method, and subsequently divided into two equal partitions.

A Nuclear genes

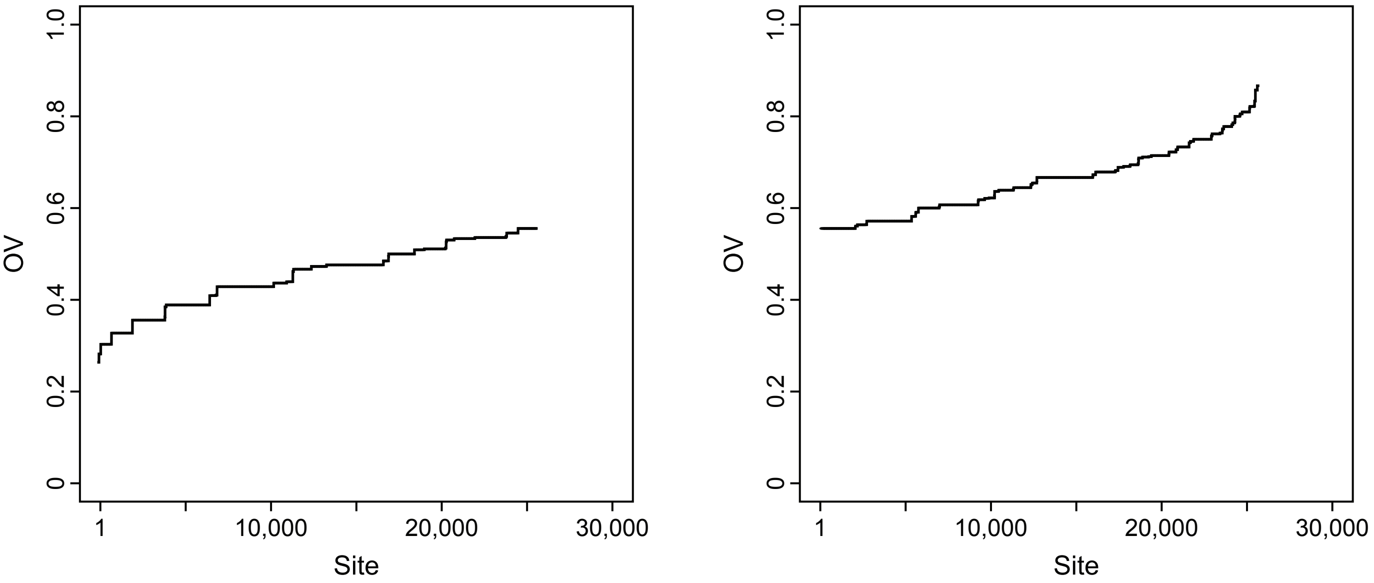

B Plastid genes

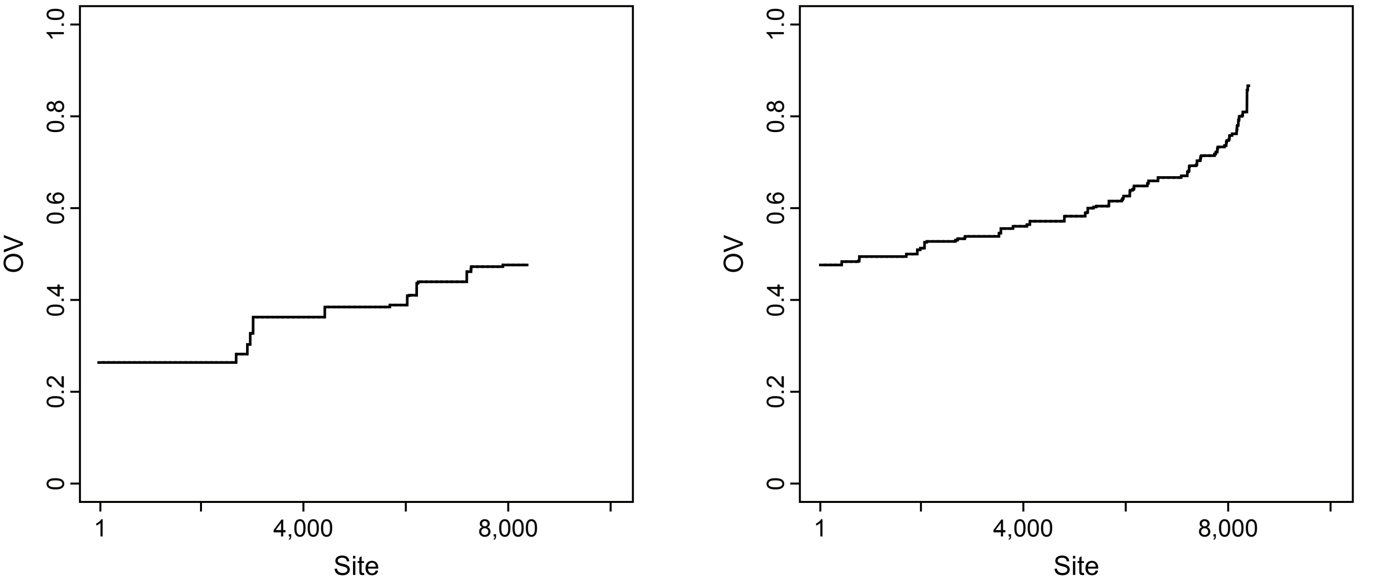

C Zhong et al. (2011)

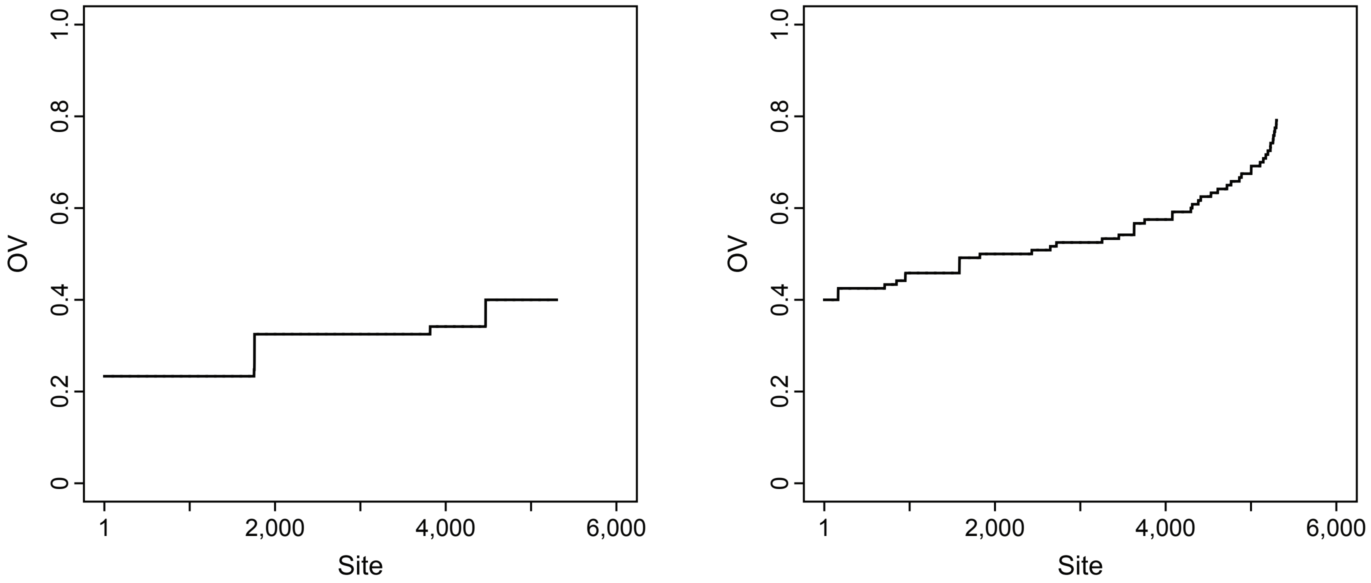

D Wu et al. (2013)

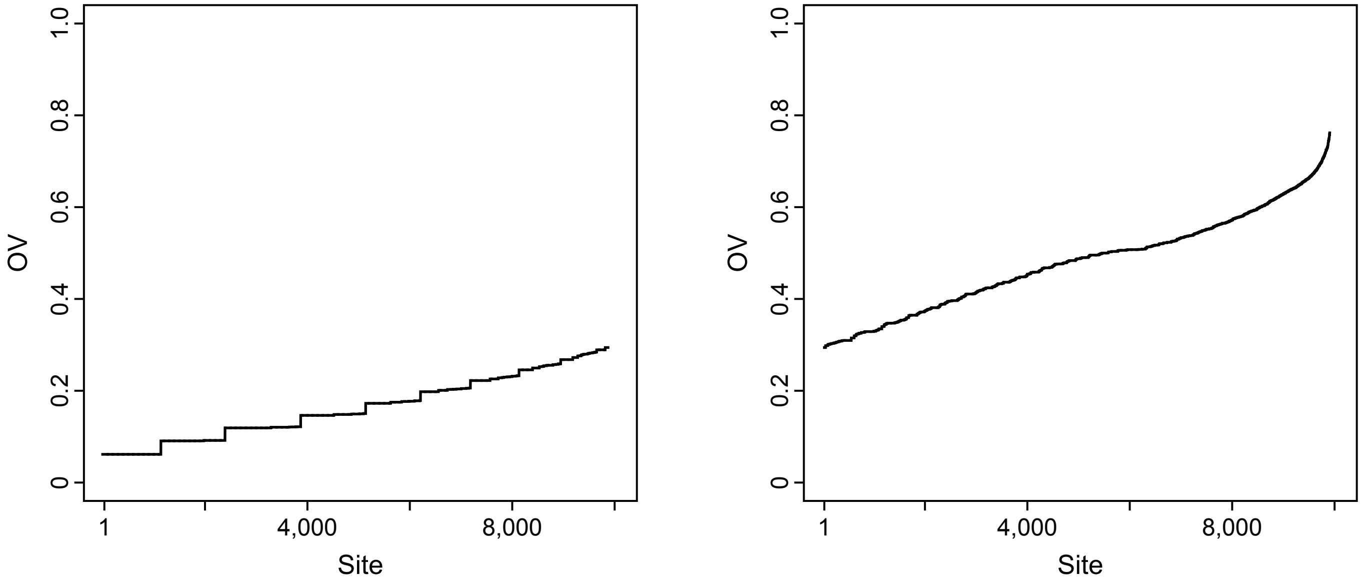

E Qiu et al. (2006)

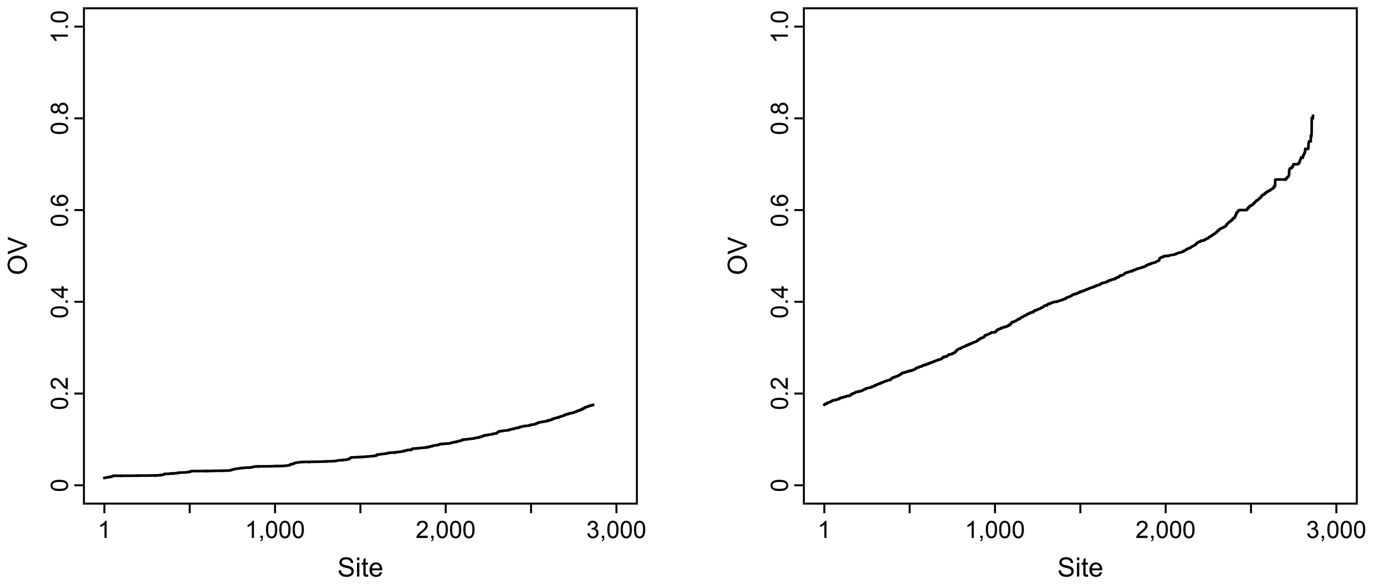

Supplement: Figure S1 — The estimated evolutionary rates for nucleotide sites in all five concatenated matrices analyzed in this study. Parsimony informative sites in each concatenated matrix were sorted based on the Observed Variability (OV) method, and subsequently divided into two equal partitions. (PDF) [file pone.0080870.s001.pdf]
