# Supplementary material for: Phylogenomics and Coalescent Analyses Resolve Extant Seed Plant Relationships
Source: PLoS One. 2013 Nov 21;8(11):e80870. doi: 10.1371/journal.pone.0080870 (PMC3836751; doi:10.1371/journal.pone.0080870)
Supplement: Table S1 — Data characteristics for all 305 nuclear genes, including the locus ID of sequence from Selaginella moellendorffii in each gene, number of species per gene, number of nucleotide sites per gene, and percentage of gaps per gene. (PDF) [file pone.0080870.s002.pdf]

Table S1. Data characteristics for all 305 nuclear genes, including the locus ID of sequence from *Selaginella moellendorffii* in each gene, number of species per gene, number of nucleotide sites per gene, and percentage of gaps per gene.

Gene cluster	*Selaginella* ID	No. of species	No. of sites	Gaps%
Cluster432	139397	12	342	0.7%
Cluster510	133023	14	333	0.6%
Cluster697	271409	11	609	0.1%
Cluster802	172404	12	321	0.1%
Cluster831	231474	10	579	0.2%
Cluster993	270704	12	351	0.4%
Cluster1015	164298	12	405	1.9%
Cluster1118	169783	12	375	0.2%
Cluster1153	147110	12	411	0.5%
Cluster1233	94273	8	663	0.6%
Cluster1249	270489	10	624	6.4%
Cluster1288	169212	13	462	0.0%
Cluster1297	133099	10	477	0.7%
Cluster1301	151128	12	495	2.2%
Cluster1303	102684	8	537	1.2%
Cluster1339	146333	11	324	0.5%
Cluster1350	97742	11	510	2.7%
Cluster1388	231406	12	642	1.1%
Cluster1390	267349	10	702	0.0%
Cluster1393	79058	10	600	0.1%
Cluster1416	438896	13	687	1.5%
Cluster1418	164273	10	555	0.5%
Cluster1498	68768	9	459	3.8%
Cluster1504	403889	11	606	1.3%
Cluster1521	411065	11	324	0.5%
Cluster1561	184168	9	630	4.7%
Cluster1565	49207	9	441	2.0%
Cluster1578	227538	11	411	0.9%
Cluster1653	98161	9	462	3.5%
Cluster1666	73724	8	573	3.9%
Cluster1700	233415	12	486	3.1%
Cluster1730	180027	10	414	0.2%
Cluster1750	107381	12	492	0.7%
Cluster1755	170654	12	504	0.7%
Cluster1777	67228	7	735	2.2%
Cluster1788	141259	12	606	0.0%
Cluster1796	58495	8	597	1.5%
Cluster1807	49683	8	306	0.2%
Cluster1824	271365	11	618	0.1%
Cluster1835	236819	11	531	1.7%
Cluster1862	91887	8	525	0.8%
Cluster1869	139492	12	645	2.5%

Cluster1899	145785	10	342	0.9%
Cluster1903	73696	10	372	1.9%
Cluster1909	132775	11	363	0.5%
Cluster1944	231365	11	534	0.8%
Cluster1981	142363	8	516	5.6%
Cluster1982	89939	9	615	3.5%
Cluster2005	402529	11	483	2.5%
Cluster2011	232048	9	630	1.1%
Cluster2021	230208	11	711	0.1%
Cluster2031	439713	9	648	2.5%
Cluster2040	268686	11	603	1.5%
Cluster2046	77479	11	387	2.6%
Cluster2049	146421	8	660	1.6%
Cluster2096	84870	8	396	3.1%
Cluster2104	110648	12	483	3.4%
Cluster2136	120943	11	480	0.1%
Cluster2141	16705	7	588	0.0%
Cluster2145	171147	10	501	1.8%
Cluster2152	81087	10	375	2.6%
Cluster2154	402189	9	636	0.0%
Cluster2201	109700	10	552	0.4%
Cluster2203	88464	9	378	6.2%
Cluster2209	270490	10	546	1.5%
Cluster2211	228566	12	324	0.0%
Cluster2214	154794	10	663	2.7%
Cluster2216	155397	12	537	1.4%
Cluster2220	266789	8	549	0.0%
Cluster2222	170735	12	357	0.4%
Cluster2244	73281	9	546	3.6%
Cluster2303	152815	12	552	1.0%
Cluster2314	88342	8	546	1.0%
Cluster2318	75361	10	531	1.8%
Cluster2326	141745	8	687	4.1%
Cluster2329	67913	8	663	2.8%
Cluster2338	68710	8	495	3.2%
Cluster2345	117290	9	564	1.3%
Cluster2348	431211	11	498	0.0%
Cluster2356	59528	11	405	5.9%
Cluster2403	80728	7	393	0.3%
Cluster2404	270833	10	546	0.7%
Cluster2405	228505	11	360	5.4%
Cluster2415	235078	11	459	1.8%
Cluster2430	100915	13	438	1.1%
Cluster2438	164004	10	651	1.4%
Cluster2450	71425	7	609	0.4%
Cluster2456	37179	7	519	0.7%
Cluster2491	134406	9	555	0.2%
Cluster2518	77814	11	684	0.8%
Cluster2532	156096	11	693	3.0%

Cluster2544	438712	10	342	0.0%
Cluster2569	107348	10	627	3.7%
Cluster2597	18122	11	360	0.2%
Cluster2598	232928	10	480	0.9%
Cluster2599	110012	8	708	3.2%
Cluster2633	173688	9	531	1.6%
Cluster2635	98081	9	327	0.3%
Cluster2636	417581	8	330	3.2%
Cluster2648	420404	7	648	6.6%
Cluster2659	231938	9	645	1.4%
Cluster2684	90855	9	513	0.0%
Cluster2695	417664	8	534	1.0%
Cluster2723	78465	9	561	0.4%
Cluster2736	141370	8	564	6.3%
Cluster2740	85116	10	447	0.3%
Cluster2746	111302	9	507	2.0%
Cluster2755	165154	10	744	0.2%
Cluster2759	110731	10	489	7.0%
Cluster2772	49639	12	339	6.2%
Cluster2801	145426	7	684	1.5%
Cluster2830	272208	10	576	0.9%
Cluster2840	73953	10	645	3.0%
Cluster2887	75793	8	519	0.3%
Cluster2895	146440	8	357	1.8%
Cluster2903	59588	9	345	0.2%
Cluster2917	90693	10	804	2.7%
Cluster2921	168418	10	522	2.4%
Cluster2969	141796	8	537	0.4%
Cluster2970	92763	11	465	0.0%
Cluster2973	74617	11	534	1.1%
Cluster2988	98270	8	699	1.3%
Cluster3023	438103	8	684	1.9%
Cluster3030	98839	7	474	2.4%
Cluster3041	231600	9	774	1.7%
Cluster3043	25619	7	651	1.7%
Cluster3084	98142	10	540	1.1%
Cluster3089	411677	8	642	1.4%
Cluster3103	163852	10	714	0.9%
Cluster3131	228080	8	318	2.6%
Cluster3152	149744	9	516	4.8%
Cluster3162	105903	8	591	2.3%
Cluster3202	166200	8	663	1.1%
Cluster3222	109689	10	594	0.4%
Cluster3229	403901	11	525	1.8%
Cluster3233	235312	8	786	1.7%
Cluster3240	84283	7	714	0.8%
Cluster3244	270732	11	576	10.1%
Cluster3250	174566	12	333	1.0%
Cluster3256	82341	10	495	0.7%

Cluster3277	266777	9	570	0.4%
Cluster3291	232085	9	702	1.5%
Cluster3292	74723	7	531	0.2%
Cluster3294	269753	10	387	0.2%
Cluster3298	231364	9	585	0.4%
Cluster3306	121042	11	435	7.5%
Cluster3331	107702	7	426	1.5%
Cluster3402	409501	10	675	1.8%
Cluster3432	76729	11	321	0.4%
Cluster3436	229065	10	354	0.2%
Cluster3440	88870	9	456	4.3%
Cluster3458	185060	11	681	2.1%
Cluster3468	444262	9	621	1.6%
Cluster3473	410867	10	399	0.6%
Cluster3476	233013	11	360	0.2%
Cluster3481	146445	9	495	0.7%
Cluster3483	96865	8	612	4.4%
Cluster3495	98687	9	321	3.8%
Cluster3541	172574	11	453	0.3%
Cluster3568	106159	8	357	0.9%
Cluster3590	233015	10	309	0.2%
Cluster3637	82059	11	372	0.8%
Cluster3653	119017	8	414	0.4%
Cluster3654	92035	8	324	0.0%
Cluster3666	270683	8	522	1.4%
Cluster3682	144908	11	399	0.1%
Cluster3731	130333	11	300	0.0%
Cluster3744	94465	9	411	0.1%
Cluster3751	173532	11	342	0.9%
Cluster3777	6403	10	402	0.1%
Cluster3781	76289	8	390	0.0%
Cluster3784	423950	8	651	0.2%
Cluster3818	82834	9	588	0.0%
Cluster3820	73466	8	315	0.1%
Cluster3839	150375	10	477	7.0%
Cluster3845	175887	8	564	0.4%
Cluster3850	123780	8	894	2.6%
Cluster3857	158994	7	438	0.5%
Cluster3863	269334	9	870	2.3%
Cluster3868	227421	9	345	4.3%
Cluster3886	68863	8	471	0.6%
Cluster3932	71806	9	462	0.1%
Cluster3951	440387	11	441	3.8%
Cluster3968	69689	8	387	6.6%
Cluster3969	102574	9	474	3.7%
Cluster4003	17835	11	390	0.6%
Cluster4022	169883	8	678	2.9%
Cluster4025	89815	8	333	0.9%
Cluster4027	164290	12	321	0.4%

Cluster4039	170924	10	414	1.6%
Cluster4067	106195	7	774	0.0%
Cluster4075	80011	9	336	1.6%
Cluster4085	124507	10	612	1.1%
Cluster4088	118004	7	528	0.5%
Cluster4093	74131	9	426	2.1%
Cluster4112	113284	8	300	0.0%
Cluster4118	234665	7	357	0.0%
Cluster4127	162298	8	525	7.6%
Cluster4137	166126	8	375	0.9%
Cluster4148	88460	10	444	0.1%
Cluster4153	160456	10	411	1.0%
Cluster4155	271344	9	516	0.6%
Cluster4163	68683	9	612	3.6%
Cluster4165	142186	12	510	6.8%
Cluster4183	76878	7	705	0.0%
Cluster4210	85922	8	552	0.3%
Cluster4269	121295	10	636	2.0%
Cluster4281	85410	7	546	1.6%
Cluster4283	59108	7	378	0.0%
Cluster4294	442225	9	633	1.9%
Cluster4321	186360	9	615	0.9%
Cluster4322	83318	7	768	2.6%
Cluster4326	88718	11	489	2.1%
Cluster4328	153217	10	387	2.9%
Cluster4341	59259	9	423	1.3%
Cluster4345	442381	8	795	1.5%
Cluster4346	80529	8	753	3.3%
Cluster4367	78217	7	720	1.4%
Cluster4382	94218	9	411	0.4%
Cluster4384	93003	8	333	0.0%
Cluster4390	270403	7	777	2.1%
Cluster4393	145922	7	873	0.9%
Cluster4403	80524	9	309	0.0%
Cluster4417	98964	9	498	0.6%
Cluster4430	167008	7	492	4.4%
Cluster4434	112137	10	354	0.1%
Cluster4436	97898	9	486	0.0%
Cluster4440	170430	9	354	0.4%
Cluster4450	114010	7	672	1.4%
Cluster4452	440813	10	513	1.2%
Cluster4486	115735	7	630	4.4%
Cluster4496	143336	7	315	1.5%
Cluster4504	169962	9	477	1.8%
Cluster4511	230271	9	513	2.4%
Cluster4522	85537	8	354	0.7%
Cluster4525	440510	9	405	2.0%
Cluster4534	76023	9	642	0.0%
Cluster4576	71455	8	630	0.1%

Cluster4602	97725	8	492	0.8%
Cluster4609	148754	8	462	3.6%
Cluster4636	415639	10	426	1.9%
Cluster4646	437454	7	537	1.0%
Cluster4652	402197	7	657	1.2%
Cluster4682	182295	9	363	0.5%
Cluster4711	81108	9	573	1.2%
Cluster4724	111729	9	408	1.2%
Cluster4727	107767	8	564	1.8%
Cluster4779	92107	9	624	2.5%
Cluster4793	227238	8	537	1.3%
Cluster4794	83849	9	345	1.6%
Cluster4802	88436	10	411	1.0%
Cluster4841	75622	8	375	0.2%
Cluster4846	230282	7	552	1.1%
Cluster4876	232741	8	333	0.4%
Cluster4878	111765	9	345	2.4%
Cluster4957	166010	8	669	3.6%
Cluster4998	228161	7	549	0.7%
Cluster5033	39013	7	330	3.7%
Cluster5043	81847	8	750	4.2%
Cluster5055	170158	7	420	7.2%
Cluster5097	99470	7	399	0.1%
Cluster5119	76801	8	486	0.8%
Cluster5148	91229	8	318	0.4%
Cluster5157	96868	9	501	1.0%
Cluster5205	97641	8	513	1.3%
Cluster5209	227363	8	345	2.1%
Cluster5230	137292	8	324	0.7%
Cluster5243	94581	9	369	6.3%
Cluster5249	115557	8	633	1.9%
Cluster5283	135190	7	810	1.6%
Cluster5316	403186	7	468	0.9%
Cluster5320	440351	7	672	0.0%
Cluster5321	439257	7	510	3.9%
Cluster5361	177104	8	366	0.9%
Cluster5371	437707	8	684	1.4%
Cluster5389	89062	7	666	0.7%
Cluster5428	37829	7	423	0.3%
Cluster5463	270742	7	870	0.3%
Cluster5468	84592	8	351	1.5%
Cluster5524	79368	7	666	5.7%
Cluster5565	406972	7	330	0.3%
Cluster5667	89370	9	345	0.4%
Cluster5683	110083	9	453	1.2%
Cluster5694	88218	7	564	2.4%
Cluster5702	69496	7	306	0.0%
Cluster5751	68901	7	378	2.3%
Cluster5853	75086	7	582	0.3%

Cluster5904	90075	7	480	0.8%
Cluster5976	68680	7	345	0.6%
Cluster6001	36847	7	504	4.5%
Cluster6012	85641	7	582	4.6%
Cluster6030	441564	7	804	3.1%
Cluster6125	37056	8	462	5.5%
Cluster6182	442703	9	462	1.4%
Cluster6193	76876	8	486	1.3%
Cluster6340	271851	8	486	0.5%
Cluster6512	58377	7	486	2.0%
Cluster6630	126891	7	312	0.0%
Cluster6644	76976	7	339	0.8%
Cluster6646	78672	7	702	1.4%
Cluster6678	111449	7	453	6.7%
Cluster7100	110563	7	369	1.9%
Cluster7194	415324	7	489	0.2%
Cluster7199	73772	7	564	2.7%
Cluster7209	74394	7	348	1.1%
