# Supplementary material for: Phylogenomics and Coalescent Analyses Resolve Extant Seed Plant Relationships
Source: PLoS One. 2013 Nov 21;8(11):e80870. doi: 10.1371/journal.pone.0080870 (PMC3836751; doi:10.1371/journal.pone.0080870)
Supplement: Table S2 — Data characteristics for all 47 plastid genes, including number of species per gene, number of nucleotide sites per gene, and percentage of gaps per gene. (PDF) [file pone.0080870.s003.pdf]

Table S2. Data characteristics for all 47 plastid genes, including number of species per gene, number of nucleotide sites per gene, and percentage of gaps per gene.

Gene	No. of species	No. of sites	Gaps%
atpA	14	1,518	0.2%
atpB	14	1,473	0.4%
atpE	14	411	0.7%
atpF	14	549	0.2%
atpI	14	741	0.0%
ccsA	14	951	1.7%
chlB	9	1,536	0.1%
chlL	9	870	0.1%
chlN	9	1,386	0.6%
clpP	11	600	1.5%
matK	14	1,479	1.2%
ndhA	7	1,092	7.9%
ndhB	7	1,476	0.3%
ndhC	7	360	1.7%
ndhD	7	1,497	0.1%
ndhF	7	2,208	5.1%
ndhG	7	531	0.2%
ndhH	7	1,173	0.0%
ndhI	7	537	3.0%
ndhJ	7	477	0.4%
ndhK	7	648	1.9%
petA	13	957	0.0%
petB	14	645	0.0%
petD	14	477	0.9%
psaA	14	2,250	0.0%
psaB	14	2,202	0.0%
psbA	14	1,050	0.0%
psbB	14	1,524	0.0%
psbC	14	1,317	0.0%
psbD	14	1,005	0.0%
rbcL	14	1,425	0.0%
rpl2	14	825	1.1%
rpl14	14	363	0.0%
rpl16	14	405	0.3%

rpl22	13	426	8.5%
rpoA	14	990	2.3%
rpoB	14	3,204	0.3%
rpoC1	14	2,028	0.3%
rpoC2	14	3,513	0.9%
rps3	14	648	0.5%
rps4	14	603	1.0%
rps7	12	465	0.1%
rps8	14	384	0.0%
rps11	14	390	0.5%
rps14	9	300	0.0%
ycf3	14	507	0.2%
ycf4	14	552	0.0%
